# Prevalence of diagnosed idiopathic hypersomnia among adults in the United States 2019–2023: analysis of healthcare claims

**DOI:** 10.1093/sleepadvances/zpag011

**Published:** 2026-01-22

**Authors:** Sarah C Markt, Jed Black, Richard K Bogan, Elizabeth T Jensen, Patricia Prince, Adina Estrin, Monica Iyer, Marisa Whalen, Jessica K Alexander, Weiyi Ni, Adeniyi T Togun, David T Plante

**Affiliations:** Jazz Pharmaceuticals, Palo Alto, CA, United States; Jazz Pharmaceuticals, Palo Alto, CA, United States; Stanford University Center for Sleep Sciences and Medicine, Palo Alto, CA, United States; School of Medicine, University of South Carolina, Columbia, SC, United States; Medical University of South Carolina, Charleston, SC, United States; Bogan Sleep Consultants, LLC, Columbia, SC, United States; School of Medicine, Wake Forest University, Winston–Salem, NC, United States; Aetion, Inc., New York, NY, United States; Aetion, Inc., New York, NY, United States; Aetion, Inc., New York, NY, United States; Jazz Pharmaceuticals, Philadelphia, PA, United States; Jazz Pharmaceuticals, Palo Alto, CA, United States; Jazz Pharmaceuticals, Palo Alto, CA, United States; Jazz Pharmaceuticals, Palo Alto, CA, United States; School of Medicine and Public Health, University of Wisconsin, Madison, WI, United States

**Keywords:** idiopathic hypersomnia, retrospective studies, healthcare administrative claims, prevalence, real-world evidence

## Abstract

**Study Objectives:**

National prevalence estimates for idiopathic hypersomnia (IH) are difficult to obtain. This study estimated the diagnosed IH prevalence among US adults.

**Methods:**

Symphony Integrated Dataverse claims (01/2015–12/2023) were analyzed. Eligible patients were aged ≥18 years with at least one medical/prescription claim in the year of interest (2019–2023) and prior year. IH was defined by ≥1 medical claim with an IH diagnosis code. Prevalence was estimated among all eligible patients in two ways: annual (IH diagnoses during year of interest) and all-time (IH diagnoses looking back all-time in the database from 2015 through year of interest). Age- and sex-adjusted prevalence estimates were also calculated using the US Census Bureau.

**Results:**

Over 179, 182, 193, 205, and 198 million adults were assessed for diagnosed IH prevalence in each respective year 2019–2023. Unweighted annual prevalence of diagnosed IH from 2019 to 2023 was 12.1, 11.1, 11.0, 10.5, and 11.1 per 100 000 persons, respectively. Unweighted all-time lookback prevalence of diagnosed IH from 2019 to 2023 was 32.7, 37.3, 40.6, 43.3, and 49.0 per 100 000 persons, respectively. From 2019 to 2023, estimated standardized numbers of US adults diagnosed with IH were 30 563, 27 975, 27 859, 26 624, and 28 754 based on annual prevalence, and 82 027, 93 768, 101 766, 107 763, and 124 905 based on all-time prevalence.

**Conclusions:**

Annual prevalence estimates (i.e. proportions of individuals with diagnosed IH during each year of interest) remained consistent across the follow-up period, ranging from 10.5 to 12.1 per 100 000 persons, signifying the rarity of the diagnosis.

## Teaser

Idiopathic hypersomnia is a neurologic disorder characterized by excessive daytime sleepiness, sleep inertia, nonrestorative nighttime sleep, prolonged and unrefreshing naps, dysautonomia, and fatigue. This study provided an up-to-date evaluation of the prevalence of diagnosed idiopathic hypersomnia among US adults (aged ≥18 years) in each year from 2019 through 2023, using Symphony Integrated Dataverse administrative claims. Annual and all-time lookback windows were applied. Annual prevalence estimates were low and stable over time, ranging from 10.5 to 12.1 per 100 000 persons; all-time estimates ranged from 32.7 to 49.0 per 100 000 persons. Both sets of estimates support rare disease designation. As diagnosis of idiopathic hypersomnia can be challenging, more research is needed to inform surveillance efforts and identify the burden of disease.

## Introduction

Idiopathic hypersomnia (IH) is a neurologic sleep disorder with potentially debilitating symptoms, including excessive daytime sleepiness, severe sleep inertia, nonrestorative nighttime sleep, prolonged, unrefreshing naps, dysautonomia (e.g. headache, orthostatic disturbance, temperature dysregulation), and fatigue [1, 2]. The diagnosis of IH is based on the presence of excessive daytime sleepiness for ≥3 months; polysomnography (PSG) and Multiple Sleep Latency Test (MSLT) result not consistent with narcolepsy; and other primary medical, neurologic, and sleep disorders being ruled out as the cause of hypersomnolence [3]. IH affects many aspects of daily life. The limited studies available have reported that people with IH experience more anxiety, depressive symptoms, cognitive difficulties, and functional impairments than people without IH [4]. Social difficulties may include poor work or school performance, decreased earnings, and loss of employment [3, 4].

National estimates of the prevalence of IH are difficult to obtain because of the complexities of diagnosis. In the absence of a validated biomarker, IH may be misdiagnosed [5]. Studies in the published literature have used a variety of methods to estimate prevalence, including different case definitions, data sources, time periods (e.g. look-back periods), and calendar time, which makes study comparisons challenging [6–8]. As such, the prevalence of IH in the United States (US) adult population has been estimated within the broad range of 0.002% and 1.5% [6, 8]. One investigation using data between 2013 and 2016 observed a 32% increase in the 2-year limited-duration prevalence of IH from 7.8 to 10.3 per 100 000 persons [7]. In 2024, the Wisconsin Sleep Cohort study identified 12 cases of probable IH among 792 participants with PSG and MSLT data, resulting in an estimated prevalence of 1.5% [8].

Given the wide range of prevalence estimates in the published literature that derive from varying methodologies, in addition to the lack of recent data, the objective of this study was to provide an up-to-date evaluation of the prevalence of diagnosed IH among US adults in 2019, 2020, 2021, 2022, and 2023, with application of different prevalence definitions.

## Materials and Methods

### Database

This study used the Symphony Integrated Dataverse (IDV), a US-based, nationally representative, de-identified administrative healthcare open claims dataset that incorporates medical, hospital, and prescription claims submitted by pharmacies, hospitals, outpatient facilities, and physician practices [9]. The Symphony database includes ~20 000 health plans, over 300 million patients with healthcare claims at any given time, and ~2.1 million prescribers, including data from networks used to adjudicate the claims across a representative coverage of therapeutic areas, geographies, and payer types. The population includes both commercial and public (Medicare and Medicaid) insurance plans from across the US, as well as assistance programs and cash payers. Variables in this database include patient demographic and clinical characteristics, prescription claims, and medical claims.

### Study period

In this retrospective study, individuals with administrative claims between November 2015 and December 2023 were eligible. The analyses were completed using the Symphony dataset and updated in October 2024 to include data through December 2023.

### Case definitions

#### Definition of prevalence base population (at-risk population)

**Figure 1:**
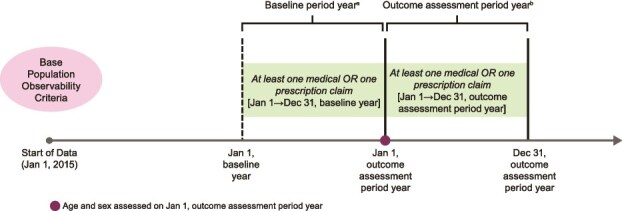
Study design, including criteria for prevalence base population. ^a^For example, for 2022 outcome assessment year, the baseline period required a medical or pharmacy claim between January 1, 2021, and December 31, 2021. ^b^For example, for 2022 outcome assessment year, a medical or pharmacy claim between January 1, 2022, and December 31, 2022.

To estimate the prevalence of diagnosed IH, this study included all adults observable in Symphony IDV (Figure 1). Individuals were considered observable if they met all the following criteria: (1) at least one medical or prescription claim between January 1 and December 31 of baseline period year (year preceding outcome assessment period year), for example, for 2022 outcome assessment period year, a medical or pharmacy claim between January 1, 2021, and December 31, 2021; (2) at least one medical or prescription claim between January 1 and December 31 of outcome assessment period year, for example, for 2022 outcome assessment period year, a medical or pharmacy claim between January 1, 2022, and December 31, 2022; (3) at least 18 years of age on January 1 of outcome assessment period year (individuals with age missing were excluded); and (4) sex of individual on record on January 1 of outcome assessment period year (individuals with missing sex were excluded).

#### Diagnosed IH case definitions

Diagnosed IH was defined as at least one medical claim (inpatient or outpatient) that included an IH diagnosis code (*International Classification of Diseases, Ninth Revision, Clinical Modification* [ICD-9-CM], 327.11, 327.12; *International Classification of Diseases, Tenth Revision, Clinical Modification* [ICD-10-CM], G47.11, G47.12). Individuals were excluded for having an inpatient or outpatient claim that included a cataplexy diagnosis code (ICD-9-CM, 347.01, 347.11; ICD-10-CM, G47.411, G47.421) on or before the date when the case definition for diagnosed IH was met. A sensitivity analysis was conducted using a case definition that included individuals with at least two inpatient or outpatient medical claims for IH on separate days; the diagnosis date was defined as the date of the second qualifying claim.

### Prevalence

Prevalence of diagnosed IH was assessed for 2019, 2020, 2021, 2022, and 2023 using annual (Figure S1A) and all-time lookback (Figure S1B) prevalences.

#### Annual period prevalence (1-year fixed-time period)

Calculation of diagnosed IH annual period prevalence for each applicable calendar year was estimated by including all individuals with diagnosed IH among all individuals who met the criteria for the prevalence base population that year (e.g. January 1, 2019–December 31, 2019, for calendar year 2019).

#### All-time lookback (overall) prevalence (extended lookback period)

The calculation of diagnosed IH prevalence for each outcome assessment period year was estimated by including all individuals with diagnosed IH from start of study data (January 1, 2015) through last day of year of interest among all eligible individuals in that outcome assessment period year (i.e. proportion of individuals who met the case definition of diagnosed IH, among all individuals who met the criteria for the prevalence base population during that period).

### Statistical analysis

Descriptive analyses were conducted, wherein demographic characteristics were summarized for the at-risk population in each outcome assessment year. Sex and US region were reported as categorical variables on January 1 of each outcome assessment period year (cohort entry date). Age was reported as both continuous and categorical variables on January 1 of each outcome assessment period year. Continuous variables were reported as mean, standard deviation (*SD*), median, and interquartile range. Categorical variables were reported as counts and percentages.

#### Prevalence estimates

Prevalence estimates were calculated by dividing the number of patients meeting the criteria for diagnosis of IH by the number of patients meeting the base population criteria for each outcome assessment period year. Each prevalence estimate was reported per 100 000 persons, with a corresponding 95% confidence interval (CI) calculated with the Clopper–Pearson exact method.

#### Estimating number of US adults with prevalent diagnosed IH

US Census Bureau estimates for each corresponding year were used to estimate the number of patients diagnosed with IH in the US for each year of interest (2019, 2020, 2021, 2022, and 2023), standardized to the US population using the standardization tool in EpiSheet [10]. Age- and sex-stratified (e.g. male, 18–24 years; female, 18–24 years, etc.) prevalence estimates were calculated for 2019, 2020, 2021, 2022, and 2023, using the all-time lookback period. The US Census Bureau’s number of US adults in each age/sex stratum in each corresponding year was used as the corresponding “weight” for each stratum-specific standardized count of prevalent individuals. Each stratum-specific standardized count of prevalent individuals was estimated by multiplying the number of individuals in each stratum in the US by the prevalence within each stratum. The estimated total number of US individuals with prevalent diagnosed IH for 2019, 2020, 2021, 2022, and 2023 was calculated as the sum of each stratum-specific standardized count of prevalent individuals.

## Results

Over 179 million adults in 2019, 182 million adults in 2020, 193 million adults in 2021, 205 million adults in 2022, and 198 million adults in 2023 were eligible for assessment of diagnosed prevalence of IH (Table 1). The mean (*SD*) age of eligible adults was 50.3 (17.2) years in 2019, 50.5 (17.5) years in 2020, 50.2 (17.7) years in 2021, 50.1 (17.9) years in 2022, and 50.8 (18.0) years in 2023. The majority of eligible adults were female, comprising 58.3% in 2019, 58.0% in 2020, 57.3% in 2021, 56.9% in 2022, and 57.5% in 2023.

**Table 1:** Baseline demographic characteristics of at-risk observable population in symphony IDV in 2019, 2020, 2021, 2022, and 2023.

Observable Population	2019	2020	2021	2022	2023
**At-risk population,** ^*^ ***n***	179 869 028	182 181 809	193 908 071	205 167 970	198 292 196
**Age,** ^†^ **years**					
Mean (*SD*)	50.3 (17.2)	50.5 (17.5)	50.2 (17.7)	50.1 (17.9)	50.8 (18.0)
Median (IQR)	52.0 (36.0, 65.0)	52.0 (36.0, 66.0)	51.0 (35.0, 65.0)	51.0 (35.0, 65.0)	52.0 (35.0, 66.0)
**Sex,** ^†^ ***n* (%)**					
Female	104 916 676 (58.3)	105 751 192 (58.0)	111 136 779 (57.3)	116 706 610 (56.9)	114 096 543 (57.5)
Male	74 952 352 (41.7)	76 430 617 (42.0)	82 771 292 (42.7)	88 461 360 (43.1)	84 195 653 (42.5)
**US region,** ^†^ ***n* (%)**					
Northeast	33 282 854 (18.5)	33 625 808 (18.5)	35 639 297 (18.4)	37 361 425 (18.2)	36 032 977 (18.2)
Midwest	38 786 373 (21.6)	39 121 645 (21.5)	41 014 304 (21.2)	42 660 352 (20.8)	41 363 433 (20.9)
South	70 625 538 (39.3)	71 365 685 (39.2)	75 574 980 (39.0)	79 538 243 (38.8)	76 961 520 (38.8)
West	34 285 903 (19.1)	34 965 765 (19.2)	38 131 121 (19.7)	41 278 107 (20.1)	39 327 643 (19.8)
Other	2 003 993 (1.1)	1 979 483 (1.1)	2 061 948 (1.1)	2 188 104 (1.1)	2 155 126 (1.1)
Missing	884 367 (0.5)	1 123 423 (0.6)	1 486 421 (0.7)	2 141 739 (1.0)	2 451 497 (1.2)

IDV, Integrated Dataverse; IQR, interquartile range; *SD*, standard deviation; US, United States.

^*^Individuals were considered to be at risk if they had ≥1 medical claim in baseline period year and ≥1 medical claim in assessment year.

^†^Assessed January 1 each year.

**Figure 2:**
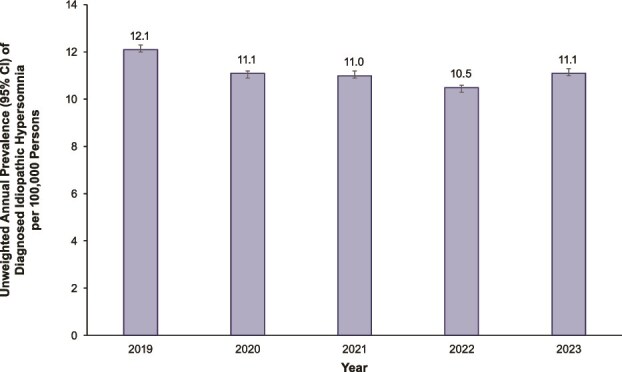
Annual prevalence^a^ of diagnosed idiopathic hypersomnia. ^a^Proportion of idiopathic hypersomnia diagnoses during year of interest. CI, confidence interval.

The annual prevalence of diagnosed IH per 100 000 persons (Figure 2) was 12.1 (95% CI, 12.0–12.3) in 2019, 11.1 (95% CI, 10.9–11.2) in 2020, 11.0 (95% CI, 10.9–11.2) in 2021, 10.5 (95% CI, 10.3–10.6) in 2022, and 11.1 (95% CI, 11.0–11.3) in 2023. The annual prevalence of diagnosed IH gradually decreased from 12.1 per 100 000 persons in 2019 to 10.5 per 100 000 persons in 2022 but increased back to 11.1 per 100 000 persons in 2023. Sensitivity analyses using at least two claims to identify IH are reported in Table S1. These estimates were lower than those using one claim and ranged from 6.9 (95% CI, 6.8–7.0) per 100 000 persons (2022) to 7.5 (95% CI, 7.4–7.7) per 100 000 persons (2023).

**Figure 3:**
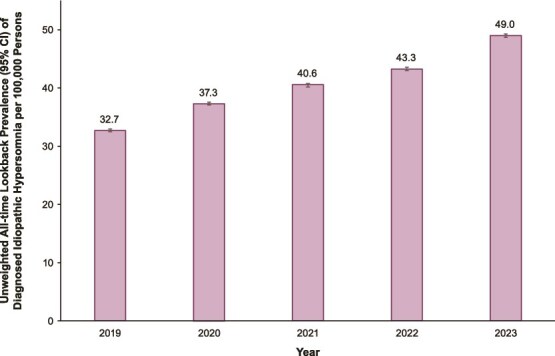
All-time lookback prevalence^a^ of diagnosed idiopathic hypersomnia. ^a^Cumulative proportion of idiopathic hypersomnia diagnoses looking back all-time in the database from January 1, 2015, through year of interest. CI, confidence interval.

The all-time lookback prevalence of diagnosed IH per 100 000 persons (Figure 3) was 32.7 (95% CI, 32.5–33.0) in 2019, 37.3 (95% CI, 37.1–37.6) in 2020, 40.6 (95% CI, 40.3–40.8) in 2021, 43.3 (95% CI, 43.0–43.6) in 2022, and 49.0 (95% CI, 48.7–49.3) in 2023. Sensitivity analyses using at least two claims to identify IH are shown in Table S1. These estimates were lower than those based on one claim and ranged from 15.2 (95% CI, 15.0–15.4) per 100 000 persons in 2019 to 24.2 (95% CI, 24.0–24.5) per 100 000 persons in 2023. For both sexes, the all-time lookback prevalence of diagnosed IH using the single claim case definition tended to increase up until the age group 34 to 44 years and then stabilized (Figure S2).

**Figure 4:**
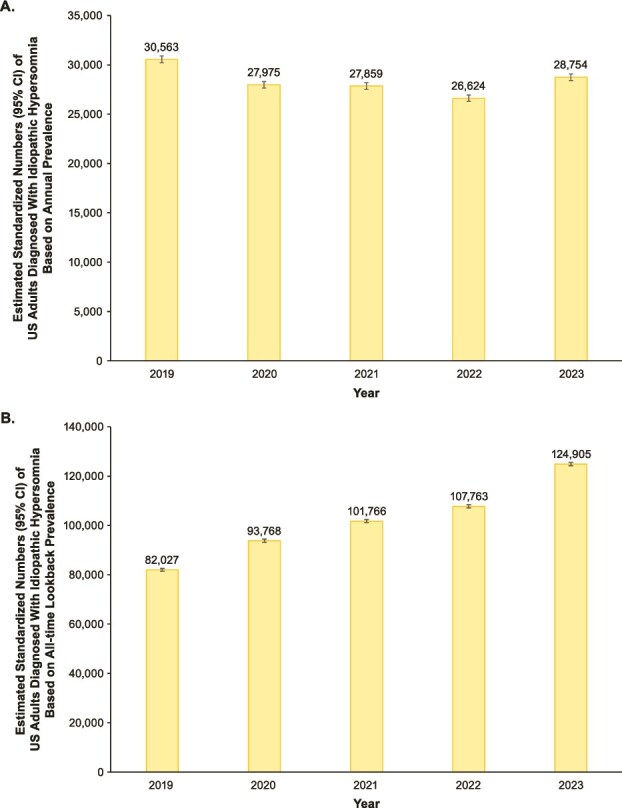
Estimated standardized numbers of US adults diagnosed with idiopathic hypersomnia based on (A) annual prevalence^a^ and (B) all-time lookback prevalence^b^. ^a^Based on annual prevalence estimates using proportion of idiopathic hypersomnia diagnoses during year of interest. ^b^Based on overall prevalence estimate using cumulative proportion of idiopathic hypersomnia diagnoses looking back all-time in the database from January 1, 2015, through year of interest. CI, confidence interval; US, United States.

Based on the annual prevalence, the estimated standardized numbers of US adults diagnosed with IH (Figure 4A) were 30 563 (95% CI, 30 221–30 908) in 2019, 27 975 (95% CI, 27 648–28 305) in 2020, 27 859 (95% CI, 27 533–28 188) in 2021, 26 624 (95% CI, 26 305–26 946) in 2022, and 28 754 (95% CI, 28 423–29 088) in 2023. The estimated standardized numbers of US adults diagnosed with IH based on the all-time lookback prevalence from 2019 to 2023 (Figure 4B) were 82 027 (95% CI, 81 467–82 590) in 2019, 93 768 (95% CI, 93 169–94 470) in 2020, 101 766 (95% CI, 101 142–102 393) in 2021, 107 763 (95% CI, 107 120–108 408) in 2022, and 124 905 (95% CI, 124 214–125 599) in 2023.

## Discussion

This study used a large US nationally representative administrative claims database to estimate the prevalence of diagnosed IH. Annual prevalence estimates of individuals diagnosed with IH remained low and relatively consistent across the follow-up period, ranging from 10.5 to 12.1 per 100 000 persons. Estimates looking back from the year of interest through all-time available in the database ranged from 0.0327% in 2019 to 0.0490% in 2023. Thus, even using the liberal single claim case definition and the more extensive lookback window yielded estimates that were consistent with rare disease designation according to the World Health Organization (diseases affecting fewer than 65 per 100 000 individuals) [11] and the US Food and Drug Administration (FDA) Orphan Drug Act (fewer than 200 000 cases in the total US population) [12]. It should be noted that these thresholds for rare disease designation concern actual or true disease prevalence in the total population, whereas the results of the current study reflect the prevalence only of diagnosed IH among individuals aged at least 18 years. However, the more sensitive case definition and longer lookback period mitigate the risk of IH underestimation and confirm the rarity of the condition.

To our knowledge, this is the most recent and comprehensive study to use real-world claims data to estimate the prevalence of diagnosed IH in the US [7]. The annual prevalence estimates reported in this study were similar to the previously reported prevalence estimates between 2013 and 2016 in US adults based on claims analyses; Acquavella et al. [7] estimated that the prevalence of IH (case definition, at least 2 IH claims) in 2016 was 10.3 per 100 000 individuals. The Wisconsin Sleep Cohort study estimated probable IH prevalence to be 1.5% (i.e. more common than previously considered) [8]; however, the study design and definition of IH were different from the present study. The Wisconsin Sleep Cohort study evaluated the prevalence of “probable” IH based on Epworth Sleepiness Scale score, PSG/MSLT findings, and exclusion of participants with obstructive sleep apnea from a cohort study of participants with available PSG and MSLT data, rather than diagnosed IH from administrative claims data [8]. Recent studies suggest that only a subset of individuals with IH actively seek medical care for their condition and, therefore, diagnosed IH prevalence estimates may understate true prevalence [13]. However, a strength of the current study was the use of an all-time lookback period, which extends prior research that typically relied on single lookback periods and provides additional opportunity to capture prevalent cases of diagnosed IH.

Prevalence estimates of diagnosed IH using the all-time lookback period were higher than estimates derived from the annual lookback periods. Given the longer observation time, all-time lookback approaches are associated with temporal trends (i.e. increasing prevalence estimates over time) [14]. It is also possible that the number of individuals diagnosed with IH in more recent years may have increased due to increased physician education and sleep expertise, more people seeking treatment for their symptoms, and the introduction of the first US FDA-approved medication for the indication [13, 15].

The present study reported two types of estimates of prevalence, including an annual and an all-time lookback period, each of which is associated with methodological pros and cons. Fixed (e.g. annual) lookbacks are more stable over time and mitigate potential sources of bias, such as differential left-censoring (i.e. variability as to when observable patients enter the dataset). For example, left-censoring may occur when a participant is included in the lookback period, but only partial information is known (e.g. the outcome is known to have taken place, but the timing of that outcome is unknown). While this type of bias can be minimized with fixed lookback periods, they also can potentially yield prevalence estimates significantly lower than those obtained with a longer lookback period [14, 16]. Since Symphony IDV is an open claims dataset, claims accrue over time and are subject to changes, as the claims have yet to be adjudicated [17, 18], which may affect fixed 1-year annual prevalence estimates to a greater extent than the all-time prevalence estimates determined using a more extensive lookback period. A possible explanation for the observed annual prevalence decreases in 2020 and prevalence increases in 2023 is the COVID-19 pandemic. Evidence generated in sleep disorders and other chronic and rare disease areas indicates a substantial decrease in diagnoses made during the pandemic [19, 20]. While the diagnostic procedures for IH and other sleep disorders are particularly ill-suited to remote management, Johnson et al. found that 90.4% of sleep centers halted or reduced in-laboratory sleep testing by at least 90% during the early pandemic [21]. Powell et al. confirm that virtually no in-laboratory sleep testing was performed in April 2020 and that such testing rebounded to only 61% of its January 2019 utilization levels by June 2021 [22]. In addition to decreased responsiveness to secular trends, such as the COVID-19 pandemic, the all-time lookback prevalence provides a comprehensive prevalence estimate that captures patients who seek care more than annually. The longer lookback period also provides a more disease-sensitive estimate, given that it includes more observable time in the database and creates more opportunity for prevalent cases to be included [14]. When codes with low sensitivity are used (e.g. codes for IH), longer lookback periods may be required to comprehensively identify the number of individuals with diagnosed IH (prevalence numerator) [5, 6]. Requiring at least two claims for the condition, rather than the single claim case definition adopted in the main analysis, reduces the likelihood of including rule-out diagnostic codes but may also have yielded artificially low prevalence estimates. In the current study, sensitivity analyses using at least two codes to identify IH showed lower estimates compared with those based on a single code. Similarly, requiring claims for PSG/MSLT in the case definition may be too restrictive and fail to capture individuals with diagnosed IH. Future research should prioritize validating an algorithm to identify true cases of IH from administrative data. Despite this inherent uncertainty, a strength of this study lies in its longitudinal evaluation of two types of prevalence estimates across the 2019–2023 study observation period.

This study was based on an analysis of medical and prescription claims used primarily for billing purposes. While claims data are valuable sources for examination of healthcare outcomes, treatment patterns, and utilization, there are limitations inherent to all claims datasets. Both overestimation and underestimation of prevalence are possible when estimates are derived from open claims. The Symphony IDV dataset did not include an enrollment file; therefore, observability criteria were applied, approximated based on healthcare utilization claims. These criteria excluded individuals from the study, specifically those with low healthcare utilization, which may have skewed the study population toward individuals with more frequent healthcare utilization and therefore potentially sicker patients who are more likely to be diagnosed with IH, potentially leading to higher prevalence estimates and impacting generalizability. Further, during the adjudication process, some cases may be redefined, resulting in duplicate resolved claims, yielding an overestimate. With open claims data, there is also, however, a potential for duplication of covered lives among insurers, as people who switch insurers midyear may be counted twice, leading to overestimation of the denominator and underestimation of prevalence [18]. Additionally, the Symphony IDV dataset lacked detailed, potentially relevant, demographic and patient characteristic data, so the study was unable to assess and report on individuals’ race, ethnicity, insurance type, socioeconomic status, provider specialty, practice setting, and laboratory and testing results.

An additional limitation of the study was the lack of a validated algorithm in claims data to identify individuals diagnosed with IH. Provider coding practices and incorrect coding in patient records, poor test–retest reliability of the MSLT for diagnosing IH, and use of one diagnosis code for IH in claims data, are potential causes of misclassification bias, resulting in overestimation or underestimation of diagnosed disease prevalence. Diagnoses that were not captured in claims were not observable in the claims dataset. While individuals with cataplexy were excluded from the definition of IH, those with diagnosis codes for other sleep disorders, including narcolepsy type 2, were not excluded from the study population, potentially resulting in misclassification and overestimation of the prevalence. Thus, these limitations listed above potentially resulted in an underestimate or overestimate of the true prevalence of IH. Despite these limitations, the prevalence estimates presented herein were generated systematically and complementary to methods described in published literature.

## Conclusion

This study is one of the most recent, thorough investigations of the prevalence of diagnosed IH among US adults. This study’s annual prevalence findings of diagnosed IH were low and relatively stable over time, consistent with previously reported annual prevalence estimates of IH in the US adult population. When using a longer lookback period, prevalence estimates were higher (although still supporting rare disease designation), highlighting important methodological considerations in estimating the burden of disease in the US population. Clinicians should be mindful in surveilling for IH to reduce patient burden associated with delayed or missed diagnosis of the disease.

## Supplementary Material

IH_Prevalence_MAN-R1-SUPPLEMENTAL_MATERIAL_CLEAN_zpag011

## Data Availability

All relevant data are provided with the manuscript and supporting files.
