# Supplementary material for: Prevalence of diagnosed idiopathic hypersomnia among adults in the United States 2019–2023: analysis of healthcare claims
Source: Sleep Adv. 2026 Jan 22;7(1):zpag011. doi: 10.1093/sleepadvances/zpag011 (PMC12978637; doi:10.1093/sleepadvances/zpag011)
Supplement: IH_Prevalence_MAN-R1-SUPPLEMENTAL_MATERIAL_CLEAN_zpag011 [file ih_prevalence_man-r1-supplemental_material_clean_zpag011.docx]

# Prevalence of Diagnosed Idiopathic Hypersomnia Among Adults in the United States 2019–2023: Analysis of Healthcare Claims

Sarah C. Markt^1^; Jed Black^1,2^;
Richard K. Bogan^3,4,5^; Elizabeth T. Jensen^6^;
Patricia Prince^7^; Adina Estrin^7,*^; Monica Iyer^7,*^; Marisa Whalen^8^; Jessica K. Alexander^1^; Weiyi Ni^1^; Adeniyi T. Togun^1^; David T. Plante^9^

^1^Jazz Pharmaceuticals, Palo Alto, CA, USA; ^2^Stanford University Center for Sleep Sciences and Medicine, Palo Alto, CA, USA; ^3^University of South Carolina School of Medicine, Columbia, SC, USA; ^4^Medical University of South Carolina, Charleston, SC, USA; ^5^Bogan Sleep Consultants, LLC, Columbia, SC, USA; ^6^Wake Forest University School of Medicine, Winston–Salem, NC, USA; ^7^Aetion, Inc., New York, NY, USA; ^8^Jazz Pharmaceuticals, Philadelphia, PA, USA; ^9^University of Wisconsin School of Medicine and Public Health, Madison, WI, USA. *Monica Iyer and Adina Estrin are former employees of Aetion.

# Supplemental Material

**Figure S1**. Definitions of (A) Annual Prevalence and (B) All-Time Lookback Prevalence

**A)
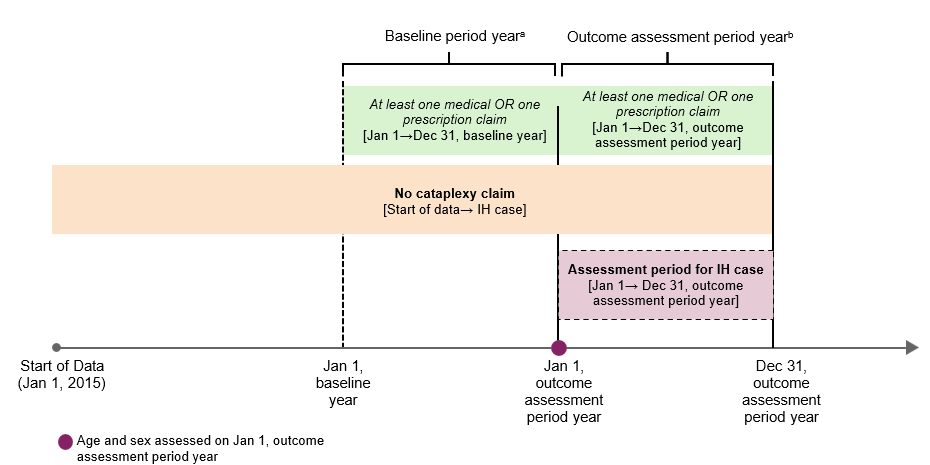
**

**B)**

**
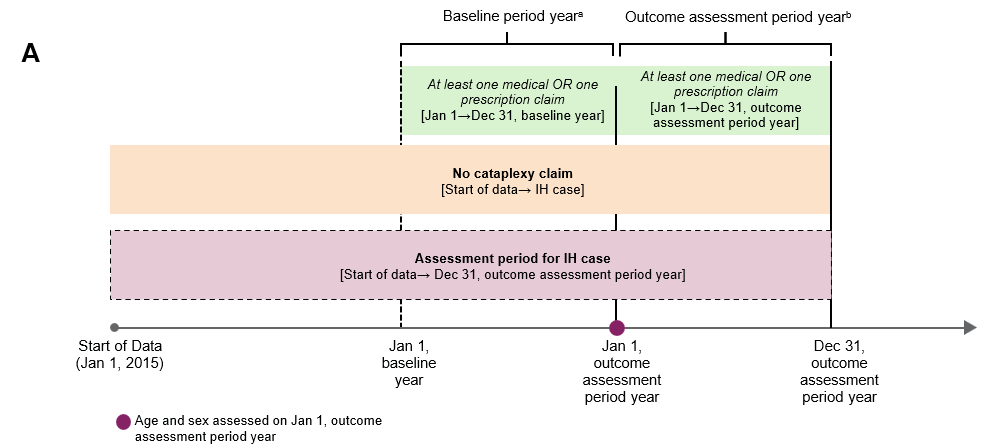
**

^a^For example, for 2022 outcome assessment year, the baseline period required a medical or pharmacy claim between January 1, 2021, and December 31, 2021.

^b^For example, for 2022 outcome assessment year, a medical or pharmacy claim between January 1, 2022, and December 31, 2022.

Dec, December; IH, idiopathic hypersomnia; Jan, January.

**Table S1.** Annual Prevalence^a^ and All-time Lookback Prevalence^b^ of Diagnosed Idiopathic Hypersomnia Using ≥2 Claims to Identify Cases

| Year | Annual Prevalence  per 100,000 Persons (95% CI) | All-time Lookback Prevalence  per 100,000 Persons (95% CI) |
| --- | --- | --- |
| 2019 | 7.3 (7.2–7.5) | 15.2 (15.0–15.4) |
| 2020 | 7.4 (7.2–7.5) | 17.8 (17.6–18.0) |
| 2021 | 7.2 (7.1–7.3) | 19.5 (19.3–19.7) |
| 2022 | 6.9 (6.8–7.0) | 21.0 (20.8–21.2) |
| 2023 | 7.5 (7.4–7.7) | 24.2 (24.0–24.5) |

^a^Proportion of idiopathic hypersomnia diagnoses during year of interest.

^b^Cumulative proportion of idiopathic hypersomnia diagnoses looking back all-time in the database from January 1, 2015, through year of interest.

CI, confidence interval.

**Figure S2.** All-time Lookback Prevalence^a^ of Diagnosed Idiopathic Hypersomnia, Stratified by Age in (A) Males and (B) Females

**A)**


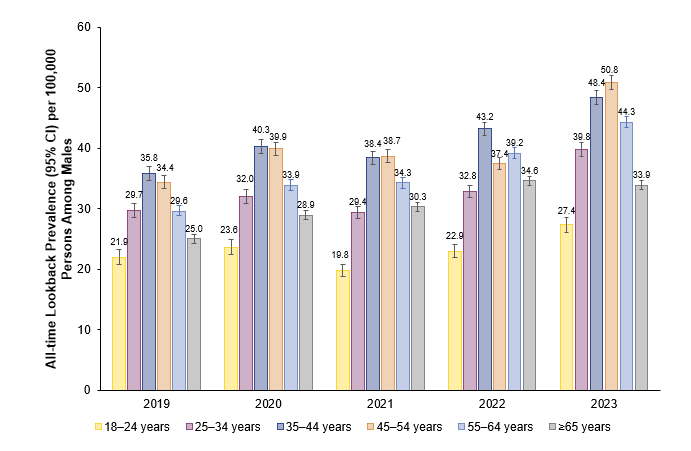


**B)**

**
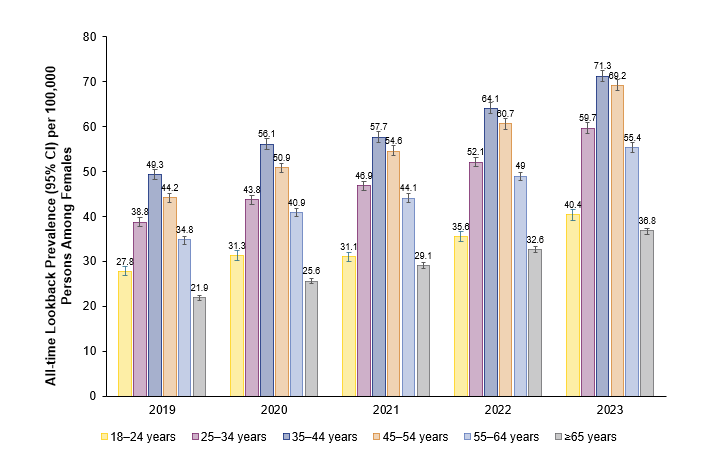
**

^a^Cumulative proportion of idiopathic hypersomnia diagnoses looking back all-time in the database from January 1, 2015, through year of interest.

CI, confidence interval.
